# Risk of thyroid cancer in relation to height, weight, and body mass index in Japanese individuals: a population‐based cohort study

**DOI:** 10.1002/cam4.1395

**Published:** 2018-03-25

**Authors:** Junya Sado, Tetsuhisa Kitamura, Tomotaka Sobue, Norie Sawada, Motoki Iwasaki, Shizuka Sasazuki, Taiki Yamaji, Taichi Shimazu, Shoichiro Tsugane, the JPHC Study Group

**Affiliations:** ^1^ Division of Environmental Medicine and Population Sciences Department of Social and Environmental Medicine Graduate School of Medicine Osaka University Osaka Japan; ^2^ Division of Epidemiology Center for Public Health Sciences National Cancer Center Tokyo Japan

**Keywords:** Body mass index, height, incidence, Thyroid cancer, weight

## Abstract

Greater height and body mass index (BMI) have been associated with an increased risk of thyroid cancer incidence in Western countries. However, few epidemiological studies have assessed the association between anthropometric factors, such as BMI, height, or weight, and thyroid cancer incidence in Asian populations. Using the population‐based Japan Public Health Center‐based prospective study database, we investigated the relationship between anthropometric factors and thyroid cancer incidence. Data on anthropometric factors were collected through a self‐administered questionnaire at baseline. The hazard ratios (HRs) and 95% confidence intervals (CIs) were calculated using the Cox proportional hazards model, and the exposure level was categorized into quintiles. A total of 49,062 men and 53,661 women enrolled between 1990 and 1994 were included in our analyses, and 191 cases (37 in men and 154 in women) of thyroid cancer were identified, with 1,695,702 person‐years of follow‐up until 2010. Compared with the male group with height ≤160 cm, HRs of the male groups with height 165–168 cm and ≥169 cm were 3.92 (95% CI; 1.33–11.55, *P* = 0.013) and 4.24 (95% CI; 1.32–13.61, *P* = 0.015), respectively, and the HR per 5‐cm increase in height was 1.12 (95% CI 1.06–1.18, *P* < 0.001). In contrast, the association between anthropometric features and the risk of thyroid cancer did not significantly differ among women. In this population, an increase in risk for increased height was observed in men, but no associations between anthropometric indexes and thyroid cancer risk were observed in women.

## Introduction

Thyroid cancer is the most common endocrine cancer, accounting for about 1% of all cancers 1, 2, and its global incidence is 1.9 per 100,000 among men and 6.1 per 100,000 among women 3. Multiple factors, including genetic and environmental influences, are associated with thyroid carcinogenesis 1, 4, 5, and well‐known risk factors for thyroid cancer are exposure to ionizing radiation in childhood 4, 6, obesity 7, 8, and diabetes 9. In addition, a meta‐analysis including Western countries and Asia also revealed an association between increased height and risk of incidence of thyroid cancer in both sexes 1, 10. However, few epidemiological studies have assessed the association between anthropometric factors such as height, weight, or body mass index (BMI) and thyroid cancer among Asian populations.

The aim of this study was to investigate the relationship between anthropometric factors and thyroid cancer incidence, using the Japan Public Health Center (JPHC) Cohort database to obtain information from a study of a large‐scale prospective, population‐based cohort with approximately 100,000 subjects. Our hypothesis was that increasing height or BMI would be associated with the risk of thyroid cancer incidence.

## Methods

### Study design, settings, and patients

The JPHC Study has been described in detail elsewhere 11, 12. In brief, this study combined two cohorts consisting of 140,420 participants (68,722 men and 71,698 women) from 11 public health centers (PHCs). We distributed a self‐administered baseline questionnaire to obtain information regarding anthropometric data, medical history, health screening, lifestyle, dietary habits, and menstrual and reproductive history (for women) from all residents aged 40–59 years in five PHC areas in 1990 (Cohort I). In addition, a similar questionnaire was distributed to all residents aged 40–69 years in six PHC areas between 1993 and 1994 (Cohort II). The JPHC Study protocol was approved by the institutional review board of the National Cancer Center, Tokyo, Japan (approval no.: 13‐021). This study was approved by the Ethical Review Board of Osaka University, Osaka, Japan.

In this study, participants from one PHC area (Katsushika, *n* = 7097) were excluded because of no data on cancer incidence. Participants were also excluded as ineligible because of non‐Japanese nationality (*n* = 51), duplicate registration (*n* = 4), incorrect date of birth (*n* = 7), emigration occurring before the start of the follow‐up period (*n* = 187), or death, moving out of a study area, or loss to follow‐up before the starting point of this study (*n* = 118). In addition, we excluded participants who did not respond to the baseline questionnaire (*n* = 26,587), those who reported a history of any type of cancer (*n* = 2148), those who failed to supply data on height or body weight (*n* = 1,134), those who supplied implausible responses for BMI (<14 kg/m^2^, or >40 kg/m^2^) (*n* = 101), and those who supplied implausible responses for height (<100 cm, or >200 cm) (*n* = 2). Finally, the number of eligible participants was 102,723 (49,062 men, 53,661 women).

### Data collection

As a baseline survey, a self‐administered questionnaire was distributed to participants in 1990 for Cohort I and in 1993–1994 for Cohort II. The questionnaire included questions about a variety of lifestyle factors including personal medical history, height, body weight, smoking habits, drinking habits, habits regarding food and beverage intake, physical activity, and reproductive and menstrual factors. With regard to height and body weight, previous studies of the JPHC had compared self‐reported BMI and measured BMI from health checkups using available data and found a very strong correlation between the two (Spearman's correlation coefficient of 0.89 in men and 0.90 in women) 13, 14.

### Follow‐up

In this study, subjects were followed from the date of responding to the baseline questionnaires until 31 December 2009, in Osaka PHC and 31 December 2010, in the other PHCs. Changes in residence status, including survival, were confirmed annually through the residential registry kept in each municipality of each of the study areas; for individuals who moved out of the area, residence status was confirmed through the municipal office of the area where they had moved. Resident and death registration are required by the Family Registration Law and Basic Residential Register Law in Japan, respectively, and the registers are believed to be complete. Checkup of the resident registry is available to anyone under the Family Registration Law. Information on each cause of death was supplemented by performing a comparison against death certificate files with permission, and the cause of death was defined according to the International Classification of Disease, 10th version (ICD‐10) 15. Among the available subjects, 14,033 died, 10,185 moved out of the study areas, and 256 were lost to follow‐up within the study follow‐up period.

### Study endpoint

Cases of thyroid cancer incidence were identified through a specific cancer registry system for the JPHC Study, which was established to collect cancer incidence data on the participants living within the study area through continuous surveillance of hospital records and population‐based cancer registries. Death certificates were used as a source of supplementary information. The site and histological types of each case were coded according to the International Classification of Diseases for Oncology, third edition (ICD‐O‐3, code: C73.9) 16. The following morphology codes were used to define histologic subtypes: papillary (8050, 8260, 8340–8344, 8350, 8450), follicular (8290, 8330–8335), medullary (8345, 8510–8513), and anaplastic (8020–8021, 8031) 7, 17. The incident cases with multiple cancer sites were followed until the date of incident case of thyroid cancer. During 1,695,702 person‐years of follow‐up (mean follow‐up period: 16.5 years), we documented 191 new thyroid cancer cases (37 in men; 154 in women). Diagnosis was confirmed by histologic or cytological examination in 92.7% of cases. The distribution of histologic types was papillary carcinoma in 160 (83.8%), follicular carcinoma in 10 (5.2%), anaplastic carcinoma in 1 (0.01%), and other or unknown histologic types in 20 (10.5%).

### Statistical analysis

We conducted all analyses in this study on the basis of sex. Baseline characteristics were expressed by height category (quartile). Baseline characteristics were expressed by height category (quartile) and calculated by analysis of variance for continuous values or chi‐squared test for categorical values to compare the distribution of baseline characteristics according to the height category. The numbers of person‐years in the follow‐up period were counted from the date of responding to the baseline questionnaire to whichever of the following occurred first: date of incidence of thyroid cancer, date of moving out of study areas, date of death, or the end of the follow‐up period.

Hazard ratios (HRs) and 95% confidence intervals (CIs) for each quartile of height, body weight, or BMI and incidence of total thyroid cancer were estimated using the Cox proportional hazards model with adjustment for potential confounders as follows 17, 18, 19. As model 1, we estimated the HRs and 95% CIs adjusted for age group at baseline and PHC areas. As model 2, we estimated the HRs and 95% CIs adjusted for the following factors, which were based on previous studies: smoking habits (never, past, current), regular drinking (yes/no), leisure‐time physical activity (<1 time/week, ≥1 time/week), past history of diabetes mellitus (yes/no), intake of green tea (3–4 cups/week, 1–2 cups/day, 3–4 cups/day, ≥5 cups/day), intake of seaweed (<3 days/week, 3–4 days/week, almost daily), health screening in the previous year (yes/no), menopausal status (yes/no) (only for women), and age at menarche (≤13, 14–15, ≥16) (only for women). In model 2, weight and height were mutually adjusted. In the subgroup analysis, HRs and their 95% CIs were also calculated for papillary carcinoma. Furthermore, the subgroup analysis by menopausal status at baseline among women was estimated in consideration of the effects of female sex hormones on thyroid cancer 18, 19. Statistical analyses were conducted using STATA version 13 MP (Stata Corp., College Station, TX). All p values were two‐sided, and the significance level was set at a p value less than 0.05.

## Results

Tables 1 and 2 show baseline characteristics by quartile of height among men and women, respectively. In both sexes, study participants with greater height were more likely to be young, current smokers, regular drinkers and have a leisure‐time physical activity. In addition, among women, those with greater height were more likely to have young age at menarche and comprised a higher proportion of menopause.

**Table 1:** Baseline characteristics by height in men

	Total	Q1	Q2	Q3	Q4	*P* values^a^
Range		115–160	161–164	165–168	169–199	
Number	49,062	14,716	10,439	12,324	11,583	
Age, mean (SD)	51.6 (8.0)	54.7 (7.7)	52.1 (7.7)	50.6 (7.7)	48.4 (7.3)	<0.001
Smoking status, *n* (%)						
Nonsmoker	11,839 (24.1)	4,124 (28.0)	2,561 (24.5)	2,762 (22.4)	2,392 (20.7)	<0.001
Past smoker	11,319 (23.1)	3,401 (23.1)	2,509 (24.0)	2,915 (23.7)	2,494 (21.5)	
Current smoker	25,818 (52.6)	7,149 (48.6)	5,357 (51.3)	6,626 (53.8)	6,686 (57.7)	
Unknown	86 (0.2)	42 (0.3)	12 (0.1)	21 (0.2)	11 (0.1)	
Total	49,062 (100.0)	14,716 (100.0)	10,439 (100.0)	12,324 (100.0)	11,583 (100.0)	
Regular drinker, *n* (%)						
No	8,665 (17.7)	2,906 (19.7)	1,927 (18.5)	2,089 (17.0)	1,743 (15.0)	<0.001
Yes	33,811 (68.9)	9,569 (65.0)	7,088 (67.9)	8,671 (70.4)	8,483 (73.2)	
Unknown	6,586 (13.4)	2,241 (15.2)	1,424 (13.6)	1,564 (12.7)	1,357 (11.7)	
Total	49,062 (100.0)	14,716 (100.0)	10,439 (100.0)	12,324 (100.0)	11,583 (100.0)	
Leisure‐time physical activity, *n* (%)						
<1 time/week	34,985 (71.3)	11,128 (75.6)	7,468 (71.5)	8,705 (70.6)	7,684 (66.3)	<0.001
≥1 time/week	11,984 (24.4)	2,875 (19.5)	2,459 (23.6)	3,152 (25.6)	3,498 (30.2)	
Unknown	2,093 (4.3)	713 (4.8)	512 (4.9)	467 (3.8)	401 (3.5)	
Total	49,062 (100.0)	14,716 (100.0)	10,439 (100.0)	12,324 (100.0)	11,583 (100.0)	
History of diabetes mellitus, *n* (%)						
No	30,165 (61.5)	9,223 (62.7)	6,536 (62.6)	7,623 (61.9)	6,783 (58.6)	<0.001
Yes	3,182 (6.5)	1,017 (6.9)	731 (7.0)	795 (6.5)	639 (5.5)	
Unknown	15,715 (32.0)	4,476 (30.4)	3,172 (30.4)	3,906 (31.7)	4,161 (35.9)	
Total	49,062 (100.0)	14,716 (100.0)	10,439 (100.0)	12,324 (100.0)	11,583 (100.0)	
Green tea consumption, *n* (%)						
≤3–4 cups/week	12,577 (25.6)	3,865 (26.3)	2,702 (25.9)	3,126 (25.4)	2,884 (24.9)	<0.001
1–2 cups/day	11,508 (23.5)	3,171 (21.5)	2,393 (22.9)	2,944 (23.9)	3,000 (25.9)	
3–4 cups/day	12,859 (26.2)	3,665 (24.9)	2,675 (25.6)	3,330 (27.0)	3,189 (27.5)	
≥5 cups/day	11,686 (23.8)	3,845 (26.1)	2,584 (24.8)	2,824 (22.9)	2,433 (21.0)	
Unknown	432 (0.9)	170 (1.2)	85 (0.8)	100 (0.8)	77 (0.7)	
Total	49,062 (100.0)	14,716 (100.0)	10,439 (100.0)	12,324 (100.0)	11,583 (100.0)	
Seaweed consumption, *n* (%)						
≤2 days/week	27,994 (57.1)	8,206 (55.8)	5,897 (56.5)	6,953 (56.4)	6,938 (59.9)	<0.001
3–4 days/week	13,747 (28.0)	4,169 (28.3)	2,967 (28.4)	3,554 (28.8)	3,057 (26.4)	
Almost daily	6,875 (14.0)	2,141 (14.5)	1,486 (14.2)	1,728 (14.0)	1,520 (13.1)	
Unknown	446	200 (1.4)	89 (0.9)	89 (0.7)	68 (0.6)	
Total	49,062 (100.0)	14,716 (100.0)	10,439 (100.0)	12,324 (100.0)	11,583 (100.0)	
Health screening in previous year, *n* (%)						
No	9,603 (19.6)	2,815 (19.1)	1,930 (18.5)	2,502 (20.3)	2,356 (20.3)	<0.001
Yes	39,162 (79.8)	11,771 (80.0)	8,446 (80.9)	9,752 (79.1)	9,193 (79.4)	
Unknown	297 (0.6)	130 (0.9)	63 (0.6)	70 (0.6)	34 (0.3)	
Total	49,062 (100.0)	14,716 (100.0)	10,439 (100.0)	12,324 (100.0)	11,583 (100.0)	

Q, quartile, SD, standard deviation.

a *P*‐values were calculated by analysis of variance for continuous values or chi‐squared test for categorical values to compare the distribution of baseline characteristics according to the height category.

**Table 2:** Baseline characteristics by height in women

	Total	Q1	Q2	Q3	Q4	*P* values^a^
Range		110–148	149–152	153–156	157–198	
Number	53,661	13,727	15,733	13,478	10,723	
Age, mean (SD)	51.9 (8.0)	55.0 (7.9)	52.4 (7.8)	50.6 (7.6)	48.7 (7.4)	<0.001
Smoking status, *n* (%)						
Nonsmoker	49,041 (91.4)	12,803 (93.3)	14,503 (92.2)	12,238 (90.8)	9,497 (88.6)	<0.001
Past smoker	822 (1.5)	157 (1.1)	234 (1.5)	205 (1.5)	226 (2.1)	
Current smoker	3,582 (6.7)	698 (5.1)	927 (5.9)	987 (7.3)	970 (9.0)	
Unknown	216 (0.4)	69 (0.5)	69 (0.4)	48 (0.4)	30 (0.3)	
Total	53,661 (100.0)	13,727 (100.0)	15,733 (100.0)	13,478 (100.0)	10,723 (100.0)	
Regular drinker, *n* (%)						
No	22,094 (41.2)	5,817 (42.4)	6,738 (42.8)	5,489 (40.7)	4,050 (37.8)	<0.001
Yes	7,139 (13.3)	1,213 (8.8)	1,920 (12.2)	1,990 (14.8)	2,016 (18.8)	
Unknown	24,428 (45.5)	6,697 (48.8)	7,075 (45.0)	5,999 (44.5)	4,657 (43.4)	
Total	53,661 (100.0)	13,727 (100.0)	15,733 (100.0)	13,478 (100.0)	10,723 (100.0)	
Leisure‐time physical activity, *n* (%)						
<1 time/week	41,096 (76.6)	10,801 (78.7)	12,207 (77.6)	10,257 (76.1)	7,831 (73.0)	<0.001
≥1 time/week	10,203 (19.0)	2,224 (16.2)	2,815 (17.9)	2,669 (19.8)	2,495 (23.3)	
Unknown	2,362 (4.4)	702 (5.1)	711 (4.5)	552 (4.1)	397 (3.7)	
Total	53,661 (100.0)	13,727 (100.0)	15,733 (100.0)	13,478 (100.0)	10,723 (100.0)	
History of diabetes mellitus, *n* (%)						
No	31,240 (58.2)	8,410 (61.3)	9,467 (60.2)	7,731 (57.4)	5,632 (52.5)	<0.001
Yes	1,589 (3.0)	521 (3.8)	468 (3.0)	343 (2.5)	257 (2.4)	
Unknown	20,832 (38.8)	4,796 (34.9)	5,798 (36.9)	5,404 (40.1)	4,834 (45.1)	
Total	53,661 (100.0)	13,727 (100.0)	15,733 (100.0)	13,478 (100.0)	10,723 (100.0)	
Green tea consumption, *n* (%)						
≤3–4 cups/week	12,975 (24.2)	3,503 (25.5)	3,861 (24.5)	3,143 (23.3)	2,468 (23.0)	<0.001
1–2 cups/day	11,190 (20.9)	2,680 (19.5)	3,198 (20.3)	2,897 (21.5)	2,415 (22.5)	
3–4 cups/day	14,810 (27.6)	3,669 (26.7)	4,348 (27.6)	3,782 (28.1)	3,011 (28.1)	
≥5 cups/day	14,167 (26.4)	3,716 (27.1)	4,175 (26.5)	3,544 (26.3)	2,732 (25.5)	
Unknown	519 (1.0)	159 (1.2)	151 (1.0)	112 (0.8)	97 (0.9)	
Total	53,661 (100.0)	13,727 (100.0)	15,733 (100.0)	13,478 (100.0)	10,723 (100.0)	
Seaweed consumption, *n* (%)						
≤2 days/week	24,762 (46.1)	6,233 (45.4)	7,112 (45.2)	6,230 (46.2)	5,187 (48.4)	<0.001
3–4 days/week	18,183 (33.9)	4,735 (34.5)	5,312 (33.8)	4,640 (34.4)	3,496 (32.6)	
Almost daily	10,205 (19.0)	2,565 (18.7)	3,150 (20.0)	2,511 (18.6)	1,979 (18.5)	
Unknown	511 (1.0)	194 (1.4)	159 (1.0)	97 (0.7)	61 (0.6)	
Total	53,661 (100.0)	13,727 (100.0)	15,733 (100.0)	13,478 (100.0)	10,723 (100.0)	
Health screening in previous year, *n* (%)						
No	10,882 (20.3)	2,186 (15.9)	3,122 (19.8)	2,907 (21.6)	2,667 (24.9)	<0.001
Yes	42,437 (79.1)	11,411 (83.1)	12,510 (79.5)	10,510 (78.0)	8,006 (74.7)	
Unknown	342 (0.6)	130 (0.9)	101 (0.6)	61 (0.5)	50 (0.5)	
Total	53,661 (100.0)	13,727 (100.0)	15,733 (100.0)	13,478 (100.0)	10,723 (100.0)	
Menopausal status, *n* (%)						
No	21,646 (40.3)	3,519 (25.6)	5,839 (37.1)	6,256 (46.4)	6,032 (56.3)	<0.001
Yes	30,253 (56.4)	9,608 (70.0)	9,366 (59.5)	6,840 (50.7)	4,439 (41.4)	
Unknown	1,762 (3.3)	600 (4.4)	528 (3.4)	382 (2.8)	252 (2.4)	
Total	53,661 (100.0)	13,727 (100.0)	15,733 (100.0)	13,478 (100.0)	10,723 (100.0)	
Age at menarche, *n* (%)						
≤13	14,267 (26.6)	2,548 (18.6)	3,883 (24.7)	4,074 (30.2)	3,762 (35.1)	<0.001
14–15	23,323 (43.5)	5,481 (39.9)	6,949 (44.2)	6,068 (45.0)	4,825 (45.0)	
≥16	14,599 (27.2)	5,286 (38.5)	4,464 (28.4)	2,960 (22.0)	1,889 (17.6)	
Unknown	1,472 (2.7)	412 (3.0)	437 (2.8)	376 (2.8)	247 (2.3)	
Total	53,661 (100.0)	13,727 (100.0)	15,733 (100.0)	13,478 (100.0)	10,723 (100.0)	

Q, quartile, SD, standard deviation.

a *P*‐values were calculated by analysis of variance for continuous values or chi‐squared test for categorical values to compare the distribution of baseline characteristics according to the height category.

Table 3 shows the risk of total thyroid cancer according to anthropometric features in both sexes. In the multivariable‐adjusted model (model 2), compared with the male group with height ≤160 cm, the HRs of the male groups with height 165–168 cm and ≥169 cm were 3.92 (95% CI 1.33–11.55, *P* = 0.013) and 4.24 (95% CI 1.32–13.61, *P* = 0.015), respectively, and the HR per 5‐cm increase in height was 1.12 (95% CI 1.06–1.18, *P* < 0.001). In analysis to exclude the incidence of thyroid cancer within the first 3 years of follow‐up, a similar association was observed between greater height and incidence of thyroid cancer (data not shown). However, there were no associations between incidence of thyroid cancer and either weight or BMI. No significant associations were observed between anthropometric features and total thyroid cancer risk in women.

**Table 3:** Hazard ratios of total thyroid cancer according to anthropometric features in both sexes

	No. cases	Person‐years	Incidence	Crude HR	95%CI	*P* values	HR1	95%CI	*P* values	HR2	95%CI	*P* values
Men												
Height, quintile (cm)												
≤160	5	237,452	2.11	1.00	(Reference)		1.00	(Reference)		1.00	(Reference)	
161–164	5	169,805	2.94	1.39	0.40–4.81	0.601	1.60	0.46–5.58	0.459	1.51	0.43–5.30	0.524
165–168	14	197,846	7.08	3.36	1.21–9.32	0.020	4.23	1.49–12.01	0.007	3.92	1.33–11.55	0.013
≥169	13	181,246	7.17	3.43	1.22–9.63	0.019	4.91	1.67–14.39	0.004	4.24	1.32–13.61	0.015
per 5‐cm increase				1.10	1.04–1.15	<0.001	1.12	1.06–1.17	<0.001	1.12	1.06–1.18	<0.001
Weight, quintile (kg)												
≤57	6	204,233	2.94	1.00	(Reference)		1.00	(Reference)		1.00	(Reference)	
58–63	5	216,350	2.31	0.78	0.24–2.55	0.680	0.84	0.26–2.76	0.773	0.62	0.19–2.06	0.438
64–69	14	175,230	7.99	2.69	1.03–7.00	0.042	3.16	1.20–8.30	0.020	1.82	0.67–4.94	0.237
≥70	12	190,536	6.30	2.13	0.80–5.67	0.131	2.76	1.02–7.50	0.046	1.18	0.40–3.44	0.764
per 5‐kg increase				1.03	1.00–1.07	0.056	1.04	1.01–1.08	0.013	1.01	0.97–1.05	0.733
BMI, quintile (kg/m^2^)												
≤21.51	9	194,240	4.63	1.00	(Reference)		1.00	(Reference)		1.00	(Reference)	
21.52–23.37	11	193,708	5.68	1.21	0.50–2.93	0.667	1.23	0.51–2.98	0.643	1.20	0.49–2.90	0.688
23.37–25.27	6	201,538	2.98	0.63	0.23–1.78	0.386	0.67	0.24–1.89	0.447	0.62	0.22–1.77	0.375
≥25.28	11	196,862	5.59	1.19	0.49–2.87	0.699	1.33	0.54–3.26	0.529	1.17	0.47–2.90	0.732
per 5‐kg/m^2^ increase				0.95	0.86–1.06	0.375	0.96	0.87–1.07	0.509	0.95	0.85–1.06	0.364
Women												
Height, quintile (cm)												
≤148	37	235,738	15.70	1.00	(Reference)		1.00	(Reference)		1.00	(Reference)	
149–152	43	269,190	15.97	1.02	0.66–1.58	0.938	0.99	0.64–1.54	0.971	0.96	0.61–1.50	0.842
153–156	41	227,575	18.02	1.15	0.74–1.79	0.543	1.12	0.71–1.76	0.636	1.05	0.65–1.68	0.842
≥157	33	176,849	18.66	1.19	0.74–1.90	0.471	1.18	0.72–1.93	0.511	1.07	0.63–1.82	0.794
per 5‐cm increase				1.01	0.98–1.04	0.454	1.01	0.98–1.04	0.513	1.00	0.97–1.04	0.807
Weight, quintile (kg)												
≤49	37	245,993	15.04	1.00	(Reference)		1.00	(Reference)		1.00	(Reference)	
50–53	27	214,117	12.61	0.84	0.51–1.38	0.489	0.82	0.50–1.35	0.435	0.81	0.49–1.34	0.416
54–59	45	237,858	18.92	1.26	0.81–1.95	0.299	1.22	0.79–1.88	0.376	1.21	0.77–1.89	0.416
≥60	45	211,385	21.29	1.42	0.92–2.19	0.116	1.36	0.88–2.11	0.162	1.36	0.85–2.17	0.197
per 5‐kg increase				1.02	1.00–1.04	0.119	1.01	0.99–1.03	0.165	1.01	0.99–1.04	0.218
BMI, quintile (kg/m^2^)												
≤21.23	33	221,944	14.87	1.00	(Reference)		1.00	(Reference)		1.00	(Reference)	
21.24–23.11	36	228,454	15.76	1.06	0.66–1.70	0.804	1.04	0.65–1.67	0.874	1.03	0.64–1.65	0.911
23.12–25.31	36	230,207	15.64	1.05	0.66–1.69	0.827	1.03	0.64–1.65	0.910	1.02	0.63–1.64	0.935
≥25.32	49	228,747	21.42	1.44	0.93–2.24	0.103	1.43	0.91–2.24	0.120	1.43	0.91–2.24	0.122
per 5‐kg/m^2^ increase				1.02	0.97–1.07	0.397	1.02	0.97–1.07	0.451	1.02	0.97–1.07	0.459

BMI, body mass index; HR, hazard rate; CI, confidence interval.

HR1: Adjusted for age and public health center area.

HR2: Adjusted for age, public health center area, smoking status, alcohol drinking, leisure‐time physical activity, history of diabetes mellitus, green tea consumption, seaweed consumption, health screening in the previous year, menopausal status (only for women), and age at menarche (only for women). Weight and height were mutually adjusted.

Table 4 shows the risk of papillary carcinoma according to anthropometric features in both sexes. In the multivariable‐adjusted model (model 2), the HRs for the groups of men with height 165–168 cm and ≥169 cm with the reference group of men ≤160 cm were 3.63 (95% CI 1.09–12.13, *P* = 0.036) and 4.05 (95% CI 1.12–14.71, *P* = 0.033), respectively, and the HR per 5‐cm increase in height was 1.12 (95% CI 1.05–1.19, *P* < 0.001). In analysis to exclude the incidence of papillary thyroid cancer within the first 3 years of follow‐up, a similar association was observed between greater height and incidence of papillary thyroid cancer (data not shown). However, there was no association between incidence of thyroid cancer and either weight or BMI. Neither height nor weight nor BMI was significantly associated with increased risk of papillary carcinoma in women.

**Table 4:** Hazard ratios of papillary carcinoma according to anthropometric features in both sexes

	No. cases	Person‐years	Incidence	Crude HR	95%CI	*P* values	HR1	95%CI	*P* values	HR2	95%CI	*P* values
Men												
Height, quintile (cm)												
≤160	4	237,452	1.68	1.00	(Reference)		1.00	(Reference)		1.00	(Reference)	
161–164	5	169,805	2.94	1.74	0.47–6.48	0.409	1.97	0.52–7.39	0.317	1.81	0.48–6.90	0.382
165–168	11	197,846	5.56	3.29	1.05–10.33	0.041	3.95	1.23–12.71	0.021	3.63	1.09–12.13	0.036
≥169	11	181,246	6.07	3.62	1.15–11.35	0.028	4.78	1.45–15.76	0.010	4.05	1.12–14.71	0.033
per 5‐cm increase				1.10	1.04–1.16	<0.001	1.12	1.06–1.18	<0.001	1.12	1.05–1.19	<0.001
Weight, quintile (kg)												
≤57	6	204,233	2.94	1.00	(Reference)		1.00	(Reference)		1.00	(Reference)	
58–63	3	216,350	1.39	0.47	0.12–1.87	0.282	0.50	0.12–2.00	0.326	0.36	0.09–1.47	0.156
64–69	12	175,230	6.85	2.31	0.87–6.15	0.094	2.69	1.00–7.23	0.051	1.51	0.54–4.22	0.431
≥70	10	190,536	5.25	1.77	0.64–4.87	0.268	2.28	0.81–6.43	0.117	0.95	0.31–2.90	0.928
per 5‐kg increase				1.03	1.00–1.07	0.089	1.04	1.01–1.08	0.025	1.01	0.96–1.05	0.798
BMI, quintile (kg/m^2^)												
≤21.51	8	194,240	4.12	1.00	(Reference)		1.00	(Reference)		1.00	(Reference)	
21.52–23.37	8	193,708	4.13	0.99	0.37–2.65	0.990	1.01	0.38–2.71	0.977	0.98	0.37–2.62	0.970
23.37–25.27	5	201,538	2.48	0.60	0.19–1.82	0.363	0.64	0.21–1.96	0.432	0.60	0.19–1.84	0.369
≥25.28	10	196,862	5.08	1.22	0.48–3.09	0.676	1.43	0.56–3.67	0.457	1.26	0.48–3.27	0.637
per 5‐kg/m^2^ increase				0.94	0.83–1.05	0.269	0.95	0.84–1.07	0.406	0.94	0.83–1.06	0.294
Women												
Height, quintile (cm)												
≤148	31	235,738	13.15	1.00	(Reference)		1.00	(Reference)		1.00	(Reference)	
149–152	35	269,190	13.00	0.99	0.61–1.60	0.964	0.94	0.58–1.53	0.794	0.89	0.55–1.46	0.658
153–156	34	227,575	14.94	1.14	0.70–1.85	0.604	1.06	0.64–1.74	0.832	0.97	0.58–1.63	0.912
≥157	29	176,849	16.40	1.25	0.75–2.07	0.388	1.17	0.69–1.99	0.562	1.04	0.59–1.84	0.887
per 5‐cm increase				1.02	0.99–1.05	0.211	1.02	0.98–1.05	0.336	1.01	0.98–1.05	0.581
Weight, quintile (kg)												
≤49	30	245,993	12.20	1.00	(Reference)		1.00	(Reference)		1.00	(Reference)	
50–53	23	214,117	10.74	0.88	0.51–1.52	0.647	0.86	0.50–1.48	0.585	0.84	0.48–1.45	0.523
54–59	37	237,858	15.56	1.27	0.79–2.06	0.323	1.23	0.76–1.99	0.402	1.19	0.72–1.95	0.503
≥60	39	211,385	18.45	1.51	0.94–2.44	0.088	1.46	0.90–2.35	0.122	1.40	0.84–2.33	0.199
per 5‐kg increase				1.02	0.99–1.04	0.144	1.01	0.99–1.04	0.195	1.01	0.99–1.04	0.336
BMI, quintile (kg/m^2^)												
≤21.23	28	221,944	12.62	1.00	(Reference)		1.00	(Reference)		1.00	(Reference)	
21.24–23.11	31	228,454	13.57	1.08	0.65–1.79	0.780	1.06	0.64–1.78	0.810	1.04	0.62–1.74	0.871
23.12–25.31	30	230,207	13.03	1.03	0.62–1.73	0.904	1.03	0.61–1.72	0.922	1.01	0.60–1.70	0.964
≥25.32	40	228,747	17.49	1.39	0.85–2.25	0.186	1.42	0.87–2.32	0.166	1.40	0.85–2.29	0.183
per 5‐kg/m^2^ increase				1.01	0.96–1.06	0.608	1.01	0.96–1.07	0.597	1.01	0.96–1.07	0.620

BMI, body mass index; HR, hazard rate; CI, confidence interval.

HR1: Adjusted for age and public health center area.

HR2: Adjusted for age, public health center area, smoking status, alcohol drinking, leisure‐time physical activity, history of diabetes mellitus, green tea consumption, seaweed consumption, health screening in the previous year, menopausal status (only for women), and age at menarche (only for women). Weight and height were mutually adjusted.

The risk of total thyroid cancer according to anthropometric features by menopausal status among women is noted in Table 5. Irrespective of menopausal status, each quartile of anthropometric features was not significantly associated with increased risk of total thyroid cancer among women.

**Table 5:** Hazard ratios of total thyroid cancer according to anthropometric features in both premenopausal women and postmenopausal women

	Premenopausal women				Postmenopausal women			
	No. cases	HR	95%CI	*P* values	No. cases	HR	95%CI	*P* values
Height, quintile (cm)								
≤148	14	1.00	(Reference)		20	1.00	(Reference)	
149–152	21	0.87	0.40–1.90	0.734	20	0.81	0.38–1.71	0.583
153–156	25	1.05	0.49–2.25	0.904	16	1.03	0.46–2.26	0.951
≥157	13	0.68	0.28–1.62	0.383	17	1.58	0.68–3.66	0.288
per 5‐cm increase		1.00	0.95–1.05	0.887		1.01	0.96–1.08	0.511
Weight, quintile (kg)								
≤49	16	1.00	(Reference)		19	1.00	(Reference)	
50–53	16	1.25	0.58–2.73	0.569	11	0.72	0.31–1.69	0.455
54–59	18	1.16	0.53–2.53	0.712	23	1.15	0.55–2.43	0.707
≥60	23	1.78	0.83–3.83	0.140	20	1.13	0.51–2.50	0.766
per 5‐kg increase		1.03	0.99–1.06	0.139		1.01	0.96–1.08	0.567
BMI, quintile (kg/m^2^)								
≤21.23	17	1.00	(Reference)		14	1.00	(Reference)	
21.24–23.11	17	0.88	0.42–1.86	0.740	18	1.22	0.54–2.76	0.628
23.12–25.31	16	1.01	0.48–2.11	0.984	17	1.16	0.51–2.63	0.722
≥25.32	23	1.52	0.75–3.05	0.243	24	1.20	0.53–2.70	0.659
per 5‐kg/m^2^ increase		1.04	0.96–1.12	0.326		0.99	0.96–1.08	0.627

BMI, body mass index; HR, hazard rate; CI, confidence interval.

HR: Adjusted for age, public health center area, smoking status, alcohol drinking, leisure‐time physical activity, history of diabetes mellitus, green tea consumption, seaweed consumption, health screening in the previous year, and age at menarche.

## Discussion

Using data from a large‐scale population‐based cohort in Japan, we observed that the risk of thyroid cancer incidence among men significantly increased with increasing height. However, there were no associations between either weight or BMI and risk of thyroid cancer incidence among men. On the other hand, neither height nor weight nor BMI was associated with the risk of thyroid cancer incidence among women.

The results of this study underscored the positive association between height and thyroid cancer incidence in men, with the finding of significant increases in HRs per 5‐cm increase in height. Previous studies, conducted mainly in Europe and the United States, have shown conflicting results regarding the association between height and incidence of thyroid cancer. Some cohort studies have shown a positive association between height and thyroid cancer incidence in both sexes 20, 21, 22, whereas other cohort studies have reported no association between height and thyroid cancer incidence among either men or women 23, 24, 25. The association between height and thyroid cancer incidence could be explained partially by the effect of insulin‐like growth factor (IGF‐1). Previous studies reported a positive association between the level of IGF‐1 and the risk of thyroid cancer incidence 1, 26. Higher IGF‐1 levels promote mutation in various cell lines including thyroid cells 1, 21, 26, 27 and contribute to increased risk of cancer incidence by stimulating cell proliferation, adhesion, and migration, and by inhibiting apoptosis 1, 21, 27. Importantly, IGF‐1 plays an important role in the regulation of postnatal growth, and taller individuals have higher levels of IGF‐1 in childhood and adolescence. Thus, the risk of thyroid cancer incidence might show a particular increase in taller men 1, 26. In a previous study investigating the association between height and several cancers, the risk of cancer by height per 5 cm was highest in thyroid cancer among men 20, and thyroid cancer might be more strongly influenced by height than cancers in other sites.

Some studies have reported that the risk of thyroid cancer incidence demonstrated an increase among women with greater height in the same way as among men 20, 21, 22. On the other hand, it has also been reported that the incidence of thyroid cancer is not associated with height among women in Western countries, as our study indicated 23, 24, 25. In this study, the lack of association between height and thyroid cancer incidence in Japanese women might be explanted by lower and narrower range of height among women 13. On the other hand, the risk of thyroid cancer incidence has been found to increase with greater height among Korean women, who have heights similar to Japanese women 20. Thus, the mechanism between height and thyroid cancer incidence remains under debate, and further evaluation of anthropometric factors related to thyroid cancer incidence is required among women globally, including in Asia.

Regarding BMI, obesity was not associated with risk of thyroid cancer incidence, irrespective of sex. This result was inconsistent with previous reports including cohort studies and meta‐analysis 7, 8, 28, 29. Various hypotheses have been offered to explain the positive association between obesity and incidence of thyroid cancer. First, thyroid‐stimulating hormone (TSH) levels have been positively associated with obesity, and exposure to higher TSH levels via obesity leads to increasing risk of thyroid goiter and subsequent thyroid cancer incidence 30. Second, leptin levels were higher in patients with thyroid cancer compared to healthy subjects in a case–control study 31. Leptin levels are associated with regulation of energy balance and insulin action 32, and obesity positively affects levels of adipokines including leptin 33. Leptin has also been shown to enhance the migration of papillary thyroid cancer 34. The proportion of participants with BMI ≥30 in our study was only 3%; however, in Western countries, it has been found to be 7–12% 24, 35, 36. As the prevalence of BMI ≥30 was lower in Japanese 14 than in European 35 or American individuals 36, the effect of BMI on the risk of thyroid cancer incidence might be small in this study.

Furthermore, anthropometric factors by menopausal status were not associated with the risk of thyroid cancer incidence among women. Both epidemiological and experimental studies suggest that steroid hormones may play a role in the etiology of thyroid cancer 37, 38, 39, 40. A previous investigation in the JPHC Study exploring the association between reproductive factors and thyroid cancer reported that lower age at menarche reduced the risk of thyroid cancer among premenopausal women, and surgical menopause increased the risk of thyroid cancer among postmenopausal women 19. However, in our analyses, mean age at menarche did not differ among the height groups, and the impact of this factor was small. On the other hand, a previous study of the association between female hormones and height reported that the beginning of menstruation with secretion of estrogen could close the epiphyseal cartilage and terminate height increases 41, 42. The mechanism of the interaction between female hormones and height, as well as the effect of these factors on thyroid cancer incidence, remains unclear, and further studies will be needed to confirm the associations.

## Limitations

This study has several limitations. First, we did not measure height and weight, and the study used self‐reported questionnaires. However, self‐reported height and weight would be less affected by recall bias in our study, because the correlation between self‐reported BMI and measured BMI was strong, as described in the Methods. Second, we did not obtain information regarding exposure to external radiation, which is one of the well‐known risk factors for thyroid cancer incidence 4, 6. Third, information on socioeconomic status was not obtained from all participants. Socioeconomic status may affect the frequencies which participants receive health screening, and it could, therefore, also be linked to the detection of thyroid cancer and, thus, be a source of detection bias 18. Fourth, unknown confounding factors might affect the association between thyroid cancer incidence and anthropometric factors. Fifth, the number of cases of thyroid cancer in men was small, and results should be replicated in a larger population.

## Conclusion

In this study population, greater height was associated with an increased risk of thyroid cancer incidence in Japanese men. However, the risk of thyroid cancer was not associated with anthropometric factors in Japanese women.

## Conflict of Interest

None declared.
